# Retraction Note: TRIM29 facilitates the epithelial‑to-mesenchymal transition and the progression of colorectal cancer via the activation of the Wnt/β-catenin signaling pathway

**DOI:** 10.1186/s13046-022-02387-1

**Published:** 2022-05-19

**Authors:** Juntao Sun, Tianyu Zhang, Mengmeng Cheng, Liwen Hong, Chen Zhang, Mengfan Xie, Peijun Sun, Rong Fan, Zhengting Wang, Lei Wang, Jie Zhong

**Affiliations:** Department of Gastroenterology, Ruijin Hospital, Shanghai Jiao Tong University School of Medicine, Shanghai, 200025 China

**Retraction Note: J Exp Clin Cancer Res 38, 104 (2019)**

**https://doi.org/10.1186/s13046-019-1098-y**

The Editor-in-Chief has retracted this Article. After publication, concerns were raised regarding overlapping panels in Figs. 6d and 7c. The authors addressed this by publishing a Correction [1]. However, further overlap was subsequently identified in Fig. 2g. The authors have provided the raw data, which has confirmed that a number of the original images were mislabeled. Additionally, the same western blot loading controls are used for different cells and treatment groups in Figs. 2d and 4a.

The Editor-in-Chief therefore no longer has confidence in the presented data.

None of the authors have responded to any correspondence from the publisher about this retraction notice.
